# VIDEOCARE: Decentralised psychiatric emergency care through videoconferencing

**DOI:** 10.1186/1472-6963-12-470

**Published:** 2012-12-20

**Authors:** Marianne V Trondsen, Stein Roald Bolle, Geir Øyvind Stensland, Aksel Tjora

**Affiliations:** 1 Norwegian Centre for Integrated Care and Telemedicine (NST), University Hospital of North Norway (UNN), P.O. Box 35, Tromsø, N-9038, Norway; 2 Division of Emergency Medical Services, University Hospital of North Norway (UNN), P.O. Box 45, Tromsø, N-9038, Norway; 3 Department South, General Psychiatric Clinic, University Hospital of North Norway (UNN), P.O. Box 6124, Tromsø, N-9291, Norway; 4 Department of Sociology and Political Science, Norwegian University of Science and Technology (NTNU), Trondheim, N-7491, Norway

**Keywords:** Psychiatry, Emergency care, Videoconference, Telemedicine, Tele-psychiatry, Norway, Qualitative study

## Abstract

**Background:**

Today the availability of specialists is limited for psychiatric patients in rural areas, especially during psychiatric emergencies. To overcome this challenge, the University Hospital of North Norway has implemented a new decentralised on-call system in psychiatric emergencies, by which psychiatrists are accessible by videoconference 24/7. In September 2011, the new on-call system was established in clinical practice for patients and health staff at three regional psychiatric centres in Northern Norway. Although a wide variety of therapies have been successfully delivered by videoconference, there is limited research on the use of videoconferenced consultations with patients in psychiatric emergencies. The aim of this study is to explore the use of videoconference in psychiatric emergencies based on the implementation of this first Norwegian tele-psychiatric service in emergency care.

**Methods/design:**

The research project is an exploratory case study of a new videoconference service in operation. By applying in-depth interviews with patients, specialists and local health-care staff, we will identify factors that facilitate and hinder use of videoconferencing in psychiatric emergencies, and explore how videoconferenced consultations matter for patients, professional practice and cooperation between levels in psychiatric care. By using an on-going project as the site of research, the case is especially well-suited for generating reliable and valid empirical data.

**Discussion:**

Results from the study will be of importance for understanding of how videoconferencing may support proper treatment and high-quality health care services in rural areas for patients in psychiatric emergencies.

## Background

Mental illness has become a significant health challenge worldwide [1,2]. The need to improve mental health services to enhance treatment access and quality is therefore urgent [2,3]. Currently, shorter hospital stays and provision of mental health care on the municipal level [2,4] are supposed to meet increased demands of health services in the future, supported by better integration between levels of health care providers through the use of ICT [5-7]. Based on the aim of *“Proper treatment – at the right place and right time”,* a key step in the new Norwegian Coordination Reform is to ensure that the specialist health care services are able to apply specialised competence to a greater extent, and contribute to development of expertise in municipal health care [7].

Recruiting and maintaining a sufficient number of clinical experts outside larger cities are a challenge [8-10], and tele-psychiatry, by real-time videoconferences (VC), is supposed to provide advanced consultative services and educational initiatives to areas with lack of psychiatrists [11]. A wide variety of therapies have been successfully delivered by tele-psychiatry, addressing a broad range of diagnoses and mental health issues [3,8,9,11-14]. These applications have increased the patients’ access to therapy, increased patients’ satisfaction, saved time and reduced patients’ travel needs [3,8,9,14,15]. Exploring young people’s experiences with tele-psychiatric consultations, Boydell et al. (2010) found that videoconferencing alleviated the patients’ anxieties regarding the encounter with a psychiatrist [8]. Moreover, Greenberg et al. (2006) reported that access to psychiatric expertise reduced burden on family caregivers, whilst local service providers experienced feelings of enhanced capacity by the increased knowledge, confidence and sense of competence in assisting their clients [11].

There is, however, limited research on videoconferencing used for emergency consultations in psychiatry [16,17]. Although experience is limited, it has been stated that acute tele-psychiatry has the potential to improve patient care and reduce emergency department overcrowding [16]. It is found to be safe, reliable for treatment and diagnoses, as well as satisfactory to emergency health staff and patients treated [16,18]. In somatic emergency medicine, videoconferencing has been successfully applied for team interactions between hospitals, and for complex and time-critical medical assessment and treatment [19-21].

While much of the evidence speaks in favour of tele-psychiatry, current social research of implementation of telemedicine and e-health services suggests that experiences and expectations of interaction between users and providers of such services are complex and challenging [22-28] and need further consideration. For instance, patient satisfaction studies for telemedical applications must be regarded as only partial evidence of service quality because of patients’ often uncritical trust in services [29]. Approaches need to include patients’ and health-care professionals’ experiences, as well as clinical, social, cultural and organisational aspects of implementation of telemedicine.

In this study we explore the use of video-conferenced consultations in psychiatric emergencies, based on the implementation of the first Norwegian tele-psychiatry service in emergency care established by the University Hospital of North Norway (UNN).

## Methods/Design

### Site of research

Department South in the General Psychiatric Clinic at UNN consists of a hospital section and three regional psychiatric centres. Because of the geographical remoteness of the regional psychiatric centres, they have not succeeded in recruiting enough psychiatrists for a 24/7 robust on-call service. To overcome this challenge, Department South has, in collaboration with the Norwegian Centre for Integrated Care and Telemedicine (NST), recently implemented a new decentralised on-call system in psychiatric emergencies. Videoconference (VC) studios are installed on the three regional psychiatric centres and in the homes of the psychiatrists that participate in the on-call system. The psychiatrists are accessible for the regional centres’ ambulant psychiatric teams and acute psychiatric wards, and are able to take part in direct patient consultations by VC. In September 2011, the new on-call system was established in clinical practice for the regional psychiatric centres, and psychiatrists are now accessible by VC 24 hours a day, 7 days a week.

The purpose of the new VC on-call service is to ensure patients in the vicinity of the regional acute psychiatric wards, local access to specialist assessment. In accordance with current strategies of Northern Norway Regional Health Authority and the Ministry of Health and Care Services [30,31], the service also aims to reduce unnecessary, as well as compulsory, admissions in acute psychiatric wards, due to expectations of increased competence and collaboration between levels in health care services.

### Research questions

This study aims to explore how tele-psychiatry can be a tool to improve decision-making and treatment of patients in psychiatric emergencies, by elaborating the following qualitative aspects of acute tele-psychiatry:

1) Which factors are of importance to facilitate or hinder use of videoconferencing in psychiatric emergencies?

2) How does videoconferencing matter in psychiatric emergencies for patients and patient involvement, and how are patients’ perspectives ensured?

3) How does videoconferencing matter in psychiatric emergencies for professional practice and coordination between the various levels of mental health care provision?

4) How can use of videoconferencing stimulate the development of competence in rural psychiatric centres and primary psychiatric care over time?

### Theoretical concepts

Although this study is empirically driven, certain theories and concepts will be useful in analysing the material. *Normalization process theory*[32] is especially tailored to study success and failures in implementation processes, in which telemedicine has served as original empirical input theory development. The concept of *domestication of technology* concerns the process through which artefacts are appropriated and re-embedded in a local context when it is put to use [33]. *Patient involvement* and *empowerment*[34] are also relevant concepts in the analysis of patient experiences. These theoretical concepts are maintained and developed in the project as sensitising tools in the inductive analysis of empirical data.

### Methods and analytical approach

The research project is designed as an explorative case study [35] of the new VC service in operation. To explore patients’ and health professionals’ experiences, organisational changes and collaboration between the local and the specialist levels in mental health care delivery, a stepwise-deductive inductive (SDI) approach [36] and issue-focused analysis [37] will be applied. In this combination themes are developed inductively on basis of empirical data, and theoretical concepts are applied as sensitising concepts in later analytical stages. Because we are interested in the participants’ experiences of VC consultations in psychiatric emergencies, in-depth interviews with users are preferred for generating data on participants’ experiences of VC consultations in psychiatric emergencies. Patients, specialists, and local health personnel will be interviewed based on purposive sampling [38]. We estimate a need for 20 interviews, but the exact number of participants will be determined when VC is established thoroughly as a regular service. Recruitment of study participants was initiated in June 2012, and the interviews are expected to be completed in the mid of 2013.

The in-depth interviews focus on participants’ experiences on using VC consultations in various situations, with support of a semi-structured interview-guide [39], applying a combination of structured questions and themes emerging in an inter-subjective dialogue [40]. All the informants will be interviewed face-to-face, and the interviews will be digitally recorded, transcribed and analysed. As part of the routine in Department South, the specialists and the local health emergency staff are asked to fill-in a standard log after every VC consultation to document important factors concerning the VC consultations; date and time, the initiator of the VC consultations, participants, issues consulted, results of the consultation and satisfaction. To complement the interviews, the material from these logs will be analysed as well.

The study protocol has been approved by the Regional Committee for Medical Research Ethics in Norway. Patients will be recruited based on voluntary participation, with written informed consent. The data material will be de-personalised and securely handled according to the ethical recommendations from the Regional Committee for Medical Research Ethics in Norway.

## Discussion

This study explores whether and how the use of VC consultations can be a tool to improve decision-making and treatment of patients in acute psychiatric care. However, there are several barriers to adoption and regular use of telemedicine in emergency care, such as regulatory, financial and cultural [41]. Health care services are also shaped by traditional rules and routines, as well as division of labour [42]. The technology *per se* is not the only aspect for frequent use or non-use of telemedicine and e-health [24,42]. At our site of research, organisational changes have been implemented to facilitate the use of new technology. By exploring qualitative aspects of how VC consultations matter for patients and professional practice, the study will elaborate on clinical, cultural and organisational issues of implementation of telemedicine in psychiatric emergency care. Identifying factors that facilitate or hinder the use of videoconferencing in psychiatric emergencies, are expected to increase our knowledge on how VC consultations may be successfully implemented and organised in emergency psychiatry. Moreover, the study will analyse patients’ experiences of VC as a communication medium in psychiatric emergencies, as well as implications for professional practice, organisational changes and collaboration between levels in psychiatric health care. In all, this knowledge can be used to develop new high-quality telemedicine services to improve health care for patients in rural areas, reduce needs for unnecessary transport of patients, and strengthen the collaboration and coordination between levels in health care delivery.

## Competing interests

The authors declare that they have no competing interests.

## Authors’ contributions

MT contributed to the design of the study and drafted the manuscript. SRB, GØS and AT contributed to the design of the study and helped to draft the manuscript. All authors read and approved the final manuscript.

## Pre-publication history

The pre-publication history for this paper can be accessed here:

http://www.biomedcentral.com/1472-6963/12/470/prepub
